# Extreme Cranial Ontogeny in the Upper Cretaceous Dinosaur *Pachycephalosaurus*

**DOI:** 10.1371/journal.pone.0007626

**Published:** 2009-10-27

**Authors:** John R. Horner, Mark B. Goodwin

**Affiliations:** 1 Museum of the Rockies, Montana State University, Bozeman, Montana, United States of America; 2 Museum of Paleontology, University of California, Berkeley, California, United States of America; University of Chicago, United States of America

## Abstract

**Background:**

Extended neoteny and late stage allometric growth increase morphological disparity between growth stages in at least some dinosaurs. Coupled with relatively low dinosaur density in the Upper Cretaceous of North America, ontogenetic transformational representatives are often difficult to distinguish. For example, many hadrosaurids previously reported to represent relatively small lambeosaurine species were demonstrated to be juveniles of the larger taxa. Marginocephalians (pachycephalosaurids + ceratopsids) undergo comparable and extreme cranial morphological change during ontogeny.

**Methodology/Principal Findings:**

Cranial histology, morphology and computer tomography reveal patterns of internal skull development that show the purported diagnostic characters for the pachycephalosaurids *Dracorex hogwartsia* and *Stygimoloch spinifer* are ontogenetically derived features. Coronal histological sections of the frontoparietal dome of an adult *Pachycephalosaurus wyomingensis* reveal a dense structure composed of metaplastic bone with a variety of extremely fibrous and acellular tissue. Coronal histological sections and computer tomography of a skull and frontoparietal dome of *Stygimoloch spinifer* reveal an open intrafrontal suture indicative of a subadult stage of development. These dinosaurs employed metaplasia to rapidly grow and change the size and shape of their horns, cranial ornaments and frontoparietal domes, resulting in extreme cranial alterations during late stages of growth. We propose that *Dracorex hogwartsia*, *Stygimoloch spinifer* and *Pachycephalosaurus wyomingensis* are the same taxon and represent an ontogenetic series united by shared morphology and increasing skull length.

**Conclusions/Significance:**

*Dracorex hogwartsia* (juvenile) and *Stygimoloch spinifer* (subadult) are reinterpreted as younger growth stages of *Pachycephalosaurus wyomingensis* (adult). This synonymy reduces the number of pachycephalosaurid taxa from the Upper Cretaceous of North America and demonstrates the importance of cranial ontogeny in evaluating dinosaur diversity and taxonomy. These growth stages reflect a continuum rather than specific developmental steps defined by “known” terminal morphologies.

## Introduction

Pachycephalosaurids are a group of ornithischian dinosaurs united by the presence of cranial ornamentation and an enlarged frontoparietal dome, a unique morphology among vertebrates. The history and description of *Pachycephalosaurus* involved many prominent dinosaur paleontologists of the early 20^th^ century. A review of their early observations and taxonomy, together with the systematic paleontology of *Pachycephalosaurus* proposed in this study, is provided in Supporting Information [Text S1].

A great many papers have been written about pachycephalosaurid crania, in particular concerning the variability of the frontoparietal domes [1,2 and references cited therein], but there are few studies that have attempted to understand the development of these unusual cranial structures. Interestingly, even though ontogeny was discussed in a few of these evaluations [3]–[8], it was not considered an important variant beyond the relative inflation of the dome, apparently because of the relatively similar sizes of the different individuals from time-equivalent strata. The presumption that individuals possessing juvenile characteristics are expected to be much smaller than adults is not corroborated in hadrosaurs [9] and ceratopsian dinosaurs [10]–[12] with a sufficient sample size. Prior evaluations of cranial variation in pachycephalosaurids did not consider earlier studies that demonstrated several dinosaur taxa showed allometric growth of the skull and a significant expression of horns, domes and bony ornaments when the skulls reached approximately 50% size. Now, with a greater number of pachycephalosaurid skulls from the Upper Cretaceous of the Western Interior of North America available for study, and the use of comparative cranial morphology, histology and computer tomography, multiple lines of evidence support our alternative hypothesis that *Dracorex hogwartsia* and *Stygimoloch spinifer* represent earlier growth stages in a single taxon, *Pachycephalosaurus wyomingensis*.

Relative, or proportional growth (allometry of Huxley [13]), refers to shape change with regard to an increase in size. Brown and Schlaikjer [10] first reported this relative growth in dinosaur crania in the Cretaceous Mongolian ceratopsid *Protoceratops*. Later studies by Rozhdesvensky [14] found the phenomenon not only in *Protoceratops*, but also in the Mongolian hadrosaurid *Saurolophus* and theropod *Tarbosaurus*. Allometric growth was apparent in these taxa, in part, because of the large number of specimens representing a range of ontogenetic transitions collected from the Mongolian strata. Dinosaurs of the Upper Cretaceous of Mongolia are tremendously abundant and this region is recognized around the world as one of the planet's most fertile collecting grounds. Due to relatively lower specimen density in the Western Interior of North America, ontogenetic transformational representatives were difficult to distinguish until Dodson [9] performed statistical studies on a group of “closely related” lambeosaurine hadrosaurs from Upper Cretaceous strata in Alberta, Canada, in 1975. Dodson's study revealed that many hadrosaurids previously reported to represent relatively small lambeosaurine species were instead juveniles of the larger taxa. These lambeosaurine taxa experienced cranial allometric growth and retained juvenile characters (neoteny) until the skulls reached approximately 50% adult size. Dodson [9] compared the retarded development of the characteristic hollow narial crest in lambeosaurines with the expansion of the casque in the avian taxon *Casuarius*. Casque development begins after the cassowary skull reaches 65% to 80% adult size. Dodson showed that extended neoteny and late stage allometry increased the morphological disparity between particular growth “stages” within the lambeosaurine taxa. In 1976, Dodson [11] published a related study on *Protoceratops*, and provided statistical data to quantify the earlier qualitative observations on allometric growth in these dinosaur skulls [10], [14].

Morphogenetic and osteohistogenetic evaluations of dinosaur genera from the Upper Cretaceous Hell Creek Formation of Montana, North and South Dakota, and equivalent age sediments in adjacent states and Canadian Provinces, are providing evidence that extreme modification of dinosaur skulls occurs when these skulls approach ≥50% adult size in many dinosaur groups, particularly the Marginocephalia. Marginocephalian dinosaur growth and histology of their cranial ornamentations follow the predicted trend of osteohistogenesis. A *Triceratops* ontogenetic series described by Horner and Goodwin [15], [16] revealed major ontogenetic modifications to cranial ornamentations on various parts of the skulls. The epiparietals and episquamosals bordering the edge of the parietal-squamosal frill are initially diminutive before expanding to larger, taller equilateral triangular bones, and later in ontogeny, flatten dorsoventrally and elongate as they merge with the edge of the frill. The smallest epiparietals, episquamosals and postorbital horns reveal the youngest histological tissues. Postorbital horns that grew straight at first, arc strikingly backward in younger individuals then recurve forward in later stages of ontogeny. Forward horn orientation and expression of the largest epiparietals and episquamosals occur in crania that are about 65% the length of the largest known specimens. We confirm a similar pattern of development and osteohistogenesis in the cranial morphology of the squamosal horns, nodes and frontoparietal dome in the skulls of the pachycephalosaurids *Dracorex hogwartsia*, *Stygimoloch spinifer* and *Pachycephalosaurus wyomingensis*.

## Results and Discussion

### Comparative Cranial Morphology of *Pachycephalosaurus*, *Stygimoloch* and *Dracorex*

In the following section, we begin with *Pachycephalosaurus* and demonstrate that the nominal pachycephalosaurid genera, *Dracorex* and *Stygimoloch*, represent the same taxon at an earlier ontogenetic stage. We use comparative cranial histology and morphology to confirm how these ontogenetic transformations developed in a single taxon, *Pachycephalosaurus* (Table 1). These growth stages reflect a growth continuum rather than specific developmental steps defined by “known” terminal morphologies. This is important because currently we do not know the ultimate size or maximum age of any dinosaur species and an adult growth stage assignment purports a potentially false terminal morphological state.

**Table 1:** A growth series of *Pachycephalosaurus wyomingensis* skulls and cranial elements from oldest to youngest.

					Skull	Fp dome	Sq horn #1	Sq horn/node #2	Sq horn/node #3	Horn/node	Ontogenetic
Specimen	Original Taxon	Reference	This study	Element	Length (mm)	Length (mm)	length, max width (mm)	length, max width (mm)	length, max width (mm)	profile	stage
AMNH 1696	*Pachycephalosaurus wyomingensis*	Brown & Schlaikjer 1943	Fig. 3A,B	skull	600	360	36, 53 (R)	23, 51 (R)	31, 42 (R)	blunt	adult
							31, 49 (L)	41, 54 (L)	28, 45 (L)		
UCMP 556078	*Pachycephalosaurus wyomingensis*	This study	Fig. 3C,D	skull (cast)	460 (est)	260 (est)	np	49, 36 (R)	np	pointed	subadult
MPM 8111	*Stygimoloch spinifer*	Goodwin et al. 1998	Figs. 2, 3E,F	partial skull	np	205	80 (est)	np, 35 (L)	np, 26 (L)	?pointed	subadult
UCMP 131163	*Stygimoloch spinifer*	Goodwin et al. 1998	Fig. 1B,C	partial skull	np	230	76	32, 42 (R)	>38, 42 (R)	pointed	subadult
UCMP 119433	*Stygimoloch spinifer* (holotype)	Galton and Sues 1983	Fig. 1A	squamosal	np	np	>109	>42, 38 (L)	>47, 37 (L)	pointed	subadult
TCNI 2004.17.1	*Dracorex hogwartsia* (holotype)	Bakker et al. 2006	Fig. 3G,H	skull	420	na	71, 34 (R)	50, 32 (R)	48, 28 (R)	pointed	juvenile
							64, 33 (L)	43, 25 (L)	54, 25 (L)		

Skull length increases through ontogeny. The squamosal horns and nodal ornaments reach maximum expression in *“Stygimoloch”* with the concurrent inflation of a relatively high and narrow frontoparietal dome. Ontogenetically, the dome continues to expand and incorporate the lateral cranial elements in adults. The squamosal horns and nodes erode and form clusters of robust, blunt ornaments along the dorsolateral margins of the squamosals. The adult condition is best exemplified by AMNH 1696. A series of adult *Pachycephalosaurus* skulls confirm the cranial morphology marked by a relatively smooth, massive frontoparietal dome, lack of a parietosquamosal shelf with clusters of conical, blunt squamosal nodes that are variable in number, sometimes asymetrical but morphologically consistent between adults. See Figures 3,6 and 7.

**Figure 1:**
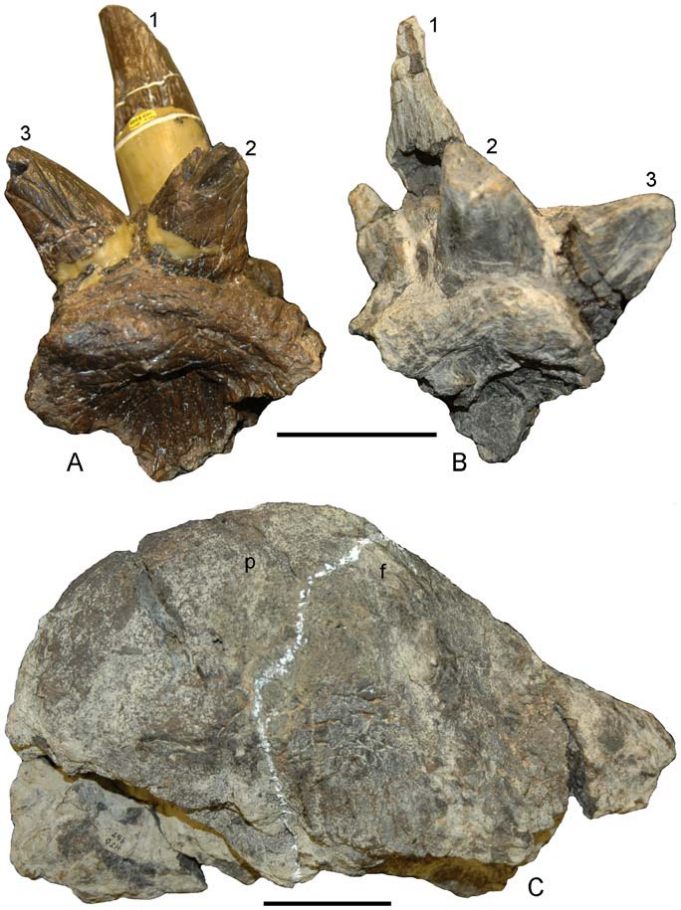
The distinctive squamosal ornamentation and relatively high, narrow frontoparietal dome of *Stygimoloch spinifer*. The holotype left squamosal (UCMP 119433) in (A) and a right squamosal (UCMP 131163) in (B) in posterior view. UCMP 131163 was found associated with the relatively high, narrow frontoparietal dome in right lateral view in (C). The frontoparietal suture is highlighted in white. The intrafrontal suture is not visible on the dorsal surface. Numbering sequence (1–3) of horns and nodes in (A) and (B) from Galton and Sues [31]. Scale bars are 5 cm.

**Figure 2:**
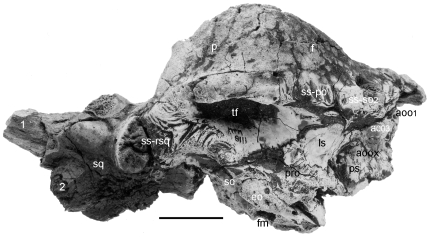
The skull of *Stygimoloch spinifer*, MPM 8111, in right lateral view. This skull revealed the low angle orientation of horn #1 and surrounding nodes on the elongated squamosal shelf. Scale bar is 5 cm.

**Figure 3:**
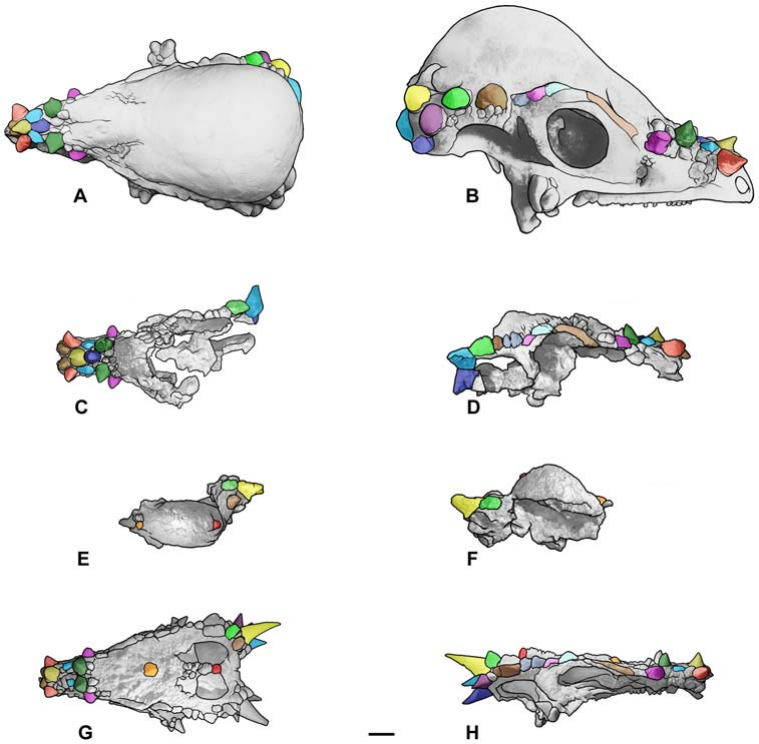
Cranial ontogenetic sequence of *Pachycephalosaurus wyomingensis* with morphological landmarks highlighted in color. The ontogenetically oldest adult, AMNH 1696, in (A) dorsal and (B) right lateral views. A younger adult, UCMP 556078 (cast) with inflation of the frontoparietal dome+lateral cranial elements and mature nasal and squamosal nodal ornamentation in (C) dorsal and (D) right lateral views. MPM 8111, a partial skull of *“Stygimoloch”* in (E) dorsal and (F) left lateral views (reversed) illustrates the high narrow frontoparietal dome, squamosal nodes and horns characteristic of the subadult growth stage. Landmarks on the dorsal skull of MPM 8111 in orange (anterior) and red (posterior) constrain the position of the dome. The youngest growth stage in this cranial ontogenetic series is “*Dracorex*”, TCNI 2004.17.1 (cast) in (G) dorsal and (H) right lateral views. The position of the squamosal horns and nasal nodes are consistent in these four pachycephalosaurid skulls, which increase in overall length and size from youngest (G,H) to oldest (A,B). Scale bar is 5 cm.

**Figure 4:**
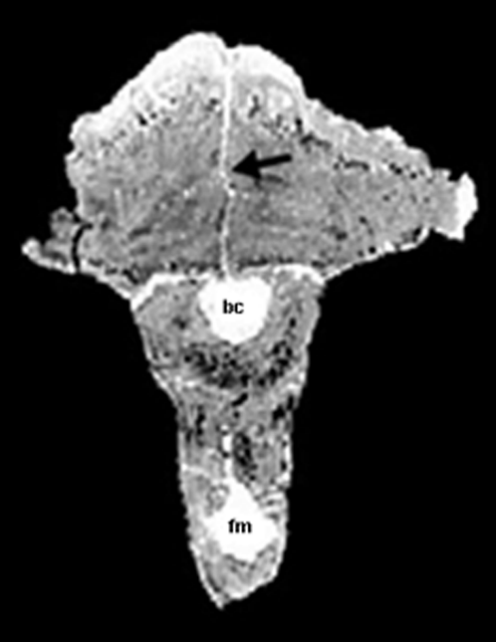
A coronal CT scan through the dome of “*Stygimoloch*” (MPM 8111). The intrafrontal suture (black arrow) is open internally supporting the subadult status of this pachycephalosaurid. The braincase (bc) and foramen magnum (fm) are clearly visible.

The frontoparietal and lateral cranial elements are highly inflated in the largest, and presumably oldest domed *Pachycephalosaurus* and follow the morphological sequence proposed for *Stegoceras* [3], [17]. The frontoparietal dome was not preserved with the isolated left squamosal and holotype of *S. spinifer*, UCMP 119433 (Figure 1A), but a dome is present in more complete pachycephalosaurid skulls with identical squamosals and squamosal horns referred to *S. spinifer* such as UCMP 131163 (Figure 1B,C) and MPM 8111 (Figure 2, Figure 3E,F). Computer tomographic analysis of MPM 8111 reveals an open intrafrontal suture internally (Figure 4) that is evidence of cranial vault expansion during postnatal craniofacial growth [18]. The intrafrontal suture functions as an intramembranous bone growth site and remains unossified as new bone is formed [18] in this subadult ontogenetic stage. The absence of a visible intrafrontal suture on the dorsal skulls of *“Stygimoloch”* previously obscured their relative age.

**Figure 5:**
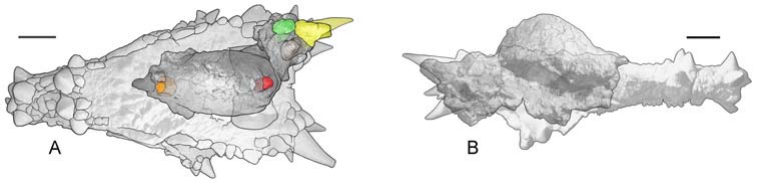
A composite image of “*Stygimoloch*” (MPM 8111) and “*Dracorex*” (TCNI 2004.17.1; cast) illustrates the subadult cranial ontogenetic stage morphology. The composite is constrained by landmarks on the skulls showing the position of the inflated frontoparietal dome of “*Stygimoloch*” on the posterior dorsal surface of “*Dracorex*” in (A) dorsal and (B) right lateral views. Scale bars are 5 cm.

In Figure 3, morphological landmarks are colored and evident on the skulls of *Pachycephalosaurus*, *Dracorex* and *Stygimoloch*. Inflation of the frontoparietal dome and corresponding closure of the temporal fenestrae are two major morphological changes that occur between *Dracorex* and *Pachycephalosaurus* with concurrent lengthening of the skull that ontogenetically separates the three pachycephalosaurids older than *“Dracorex”* shown in Figure 3A–F (see Table 1). A composite image of the holotype of *Dracorex* (TCMI 2004.17.1) and the most complete skull known of *Stygimoloch* (MPM 8111) catalogued in a repository shows the frontoparietal dome of MPM 8111 aligned with the skull of *Dracorex* (Figure 5). The composite morphology illustrates the considerable missing anterior portion of MPM 8111 that if not taken into consideration, gives the incorrect impression that this domed *“Stygimoloch”* is significantly shorter in length than *“Dracorex”*. The preserved morphology of MPM 8111 is limited to the emerging frontoparietal dome and elongated horned squamosals. Landmarks confirm the position of the incipient dome on the *“Dracorex-Stygimoloch”* morph in Figure 5. The combined cranial morphology of this subadult ontogenetic stage was verified by the authors and Sullivan [8:359] in two skulls from the Hell Creek Formation, Garfield County, Montana, that are privately held and currently unavailable for study. UCMP 556078 (Figure 3C,D) is a slightly more advanced subadult stage of ontogeny, compared to the composite skull in Figure 5, with an inflated frontoparietal and lateral elements that are enlarged but not yet incorporated into the dome as they are in *Pachycephalosaurus*. We predict this ontogenetic trend, if projected backward toward an even younger end member than *“Dracorex”*, would result in a flat-headed morph <43 cm long ( = midline length of *“Dracorex”*) with a slightly thickened (or incipiently domed) frontoparietal region, open supratemporal fenestrae and dorsal skull covered with emerging nodal ornamentation with clusters of relatively smaller diameter pyramidal nodes on the squamosals. A slightly less robust, narrower skull compared to the holotype of *“Dracorex”*, with these precise morphological characteristics, is figured in the literature [19:6] and discussed by Sullivan [8]; however it is privately held.

**Figure 6:**
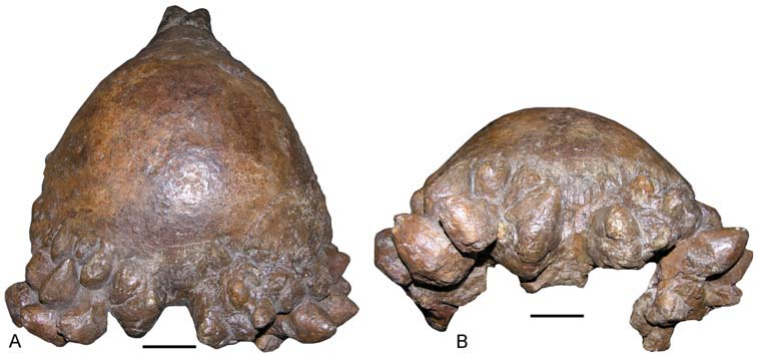
The holotype adult skull of *Pachycephalosaurus “reinheimeri”* (DMNS 469). (A) Asymmetrical clusters of massive, slightly pointed to rounded nodal ornaments on the dorsal surface of the squamosals dominate the posterior skull. (B) The squamosal nodes in posterior view. Scale bars are 5 cm.

**Figure 7:**
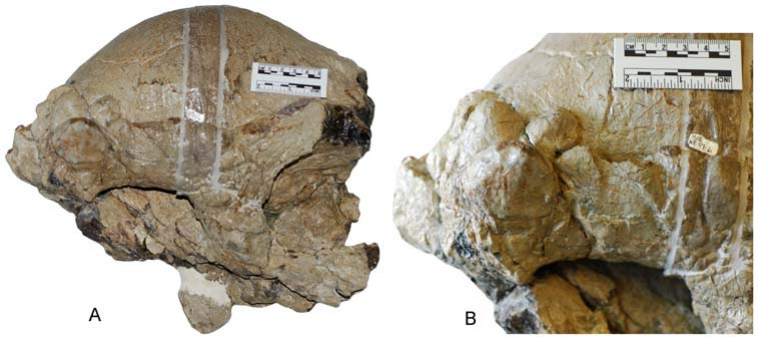
An adult *Pachycephalosaurus* skull (VRD 13) with ontogenetically advanced squamosal ornamentation. The adult cranial morphology and clusters of squamosal nodes in (A) and a close-up of the right squamosal ornamentation in (B) in right lateral views.

A series of prominent pyramidal nodes on the anterodorsal surface of the nasals in the holotype of *Dracorex hogwartsia* (Figure 3G,H) match the nasal ornaments preserved in the AMNH 1696 skull of *Pachycephalosaurus wyomingensis* (Figure 3A,B) [8] and a slightly younger *P. wyomingensis* skull (Figure 3C,D). We concur with Bakker et al. [20:331] that the “long snout, two half-rings of pyramidal spikes on the snout…” of *“Dracorex”* are nearly identical to the arrangement observed in *Pachycephalosaurus* and *“Stygimoloch”*. The distinctive cluster of squamosal horns undergoes allometric growth before they are reduced in subadults. The slightly curved squamosal horns and nodes increase in length ontogenetically from *“D.” hogwartsia* (Figure 3G,H) to *“S.” spinifer* (Figure 3E,F) before the horns erode and nodes are modified into their blunt but robust pyramidal shape in the ontogenetically most advanced skulls of *P. wyomingensis* (Figure 3A,B). The nodes form asymmetrical clusters on the dorsoposterior surface of the squamosals in the adult *Pachycephalosaurus* (Figure 6, Figure 7). Just as the number, arrangement and shape of the epiparietals and episquamosals vary between individual *Triceratops* [16], the variability in the arrangement, dimensions and symmetry of the nodal ornamentation on the posterior skull margin in *Pachycephalosaurus* is neither unexpected nor unique within the Marginocephalia [8], [21]. While a strict 1∶1 correspondence between the squamosal ornamentation in *“Dracorex”* and *Pachycephalosaurus* is not exact, it is clearer between *“Dracorex”* and *“Stygimoloch”* before the frontoparietal undergoes extreme ontogenetic modification, particularly in the posterior-most region of the skull with the expansion of the cranial vault. Individual variation within this ontogenetic continuum plays a major role in the morphology of this highly modified region of the skull. Alternatively, these “morphs” could represent different taxa, but evidence from comparative cranial histology, computer tomography, morphology, and similar patterns of growth observed in *Stegoceras* [17] and *Triceratops* [15], [16] does not support this interpretation.

Giffin's study [7] of seven pachycephalosaurids assesses geometric growth and relative frontoparietal dome shape using two landmarks restricted to the frontoparietal dome in a geographically diverse sample too small for statistical testing. Cranial ornamentation, presence or absence of morphological features and incorporation of lateral elements into the dome cannot be confirmed in this sample. Nonetheless, four specimens co-occur in the Hell Creek Formation, Carter Co., Montana. Of these, AMNH 1696 (adult *Pachycephalosaurus*), USNM 264304 (adult *Pachycephalosaurus*), and CCM 87-1 (*“Stygimoloch”*) support our ontogenetic hypothesis for *Pachycephalosaurus*. The fourth skull is *Stegoceras edmontonensis* ( = *Sphaerotholus buchholtzae* Williamson and Carr 2002; *Prenocephale edmontonensis* Sullivan 2003) and confirms these genera are sympatric in this assemblage.

### Cranial Histology

Bone histology is a valuable tool for assessing various growth parameters including relative growth rate and general bone ontogeny. Radial vascularization, for example, “…is functionally associated with very fast deposition of relatively modest amounts of new compact bone” [22:512]. Additionally, woven bone with minimal osteonal deposition is indicative of early osteogenesis, whereas dense Haversian or reconstructed tissues are indicative of later osteogenesis [17]. We utilize these general histological observations to ontogenetically order the various pachycephalosaurid cranial characteristics described in this study (Table 1, Table 2).

**Table 2:** A growth series of *Pachycephalosaurus wyomingensis* skulls and cranial elements from youngest to oldest, plus two pachycephalosaurid skulls examined in this study.

Specimen	Taxon	Reference	Type	Element	This study: Ontogenetic Series	Formation & State
					*Pachycephalosaurus wyomingensis*	
TCNI 2004.17.1	*Dracorex hogwartsia*	Bakker et al. (2006)	x	skull (cast)	juvenile	Hell Creek: SD
UCMP 119433	*Stygimoloch spinifer*	Galton & Sues (1983)	x	squamosal	subadult	Hell Creek: MT
YPM 335	*Triceratops*	Marsh (1896)		squamosal	subadult	Lance: WY
	*Triceratops*	Hatcher et al. (1907)				
	*Pachycephalosaurus* sp.	Brown & Schlaijker (1943)				
	*Stygimoloch spinifer*	Galton & Sues (1983)				
	*Dracorex hogwartsia*	Bakker et al. (2006)				
UCMP 119433	*Stygimoloch spinifer*	Galton & Sues (1983)	x	squamosal	subadult	Hell Creek: MT
MPM 7111	*Stenotholus kohleri*	Giffin et al. (1987)	x	partial skull	subadult	Hell Creek: MT
	*Stygimoloch spinifer*	Gabriel & Berghaus (1988)				
MPM 8111	*Stygimoloch spinifer*	Gabriel & Berghaus (1988)		partial skull	subadult	Hell Creek: ND
UCMP 131163	*Stygimoloch spinifer*	Goodwin et al. (1998)		partial skull	subadult	Hell Creek: MT
UCMP 128383	*Stygimoloch spinifer*	Goodwin et al. (1998)		partial skull & skeleton	subadult	Hell Creek: MT
		Goodwin & Horner (2004)				
AMNH 21542	*Stygimoloch spinifer*	Goodwin et al. (1998)		partial skull & skeleton	subadult	Hell Creek: MT
CCM 87-1	*Pachycephalosaurus wyomingensis*	Giffin (1989)		frontoparietal dome (cast)	subadult	Hell Creek: MT
MOR 560	*Pachycephalosaurus wyomingensis*	This study		squamosal horn	subadult	Hell Creek: MT
UCMP 556078	*Pachycephalosaurus wyomingensis*	This study		skull (cast)	subadult	Lance: SD
USNM 264304	*Pachycephalosaurus wyomingensis*	Giffin (1989)		frontoparietal dome	adult	Hell Creek: MT
USNM 12031	*Troödon wyomingensis*	Gilmore (1931)	x	partial skull (lit.)	adult	Lance: WY
CM 3180	*Troödon wyomingensis*	Gilmore (1936)		partial skull (lit.)	adult	Lance: WY
AMNH 1696	*Pachycephalosaurus grangeri*	Brown & Schlaikjer (1943)	x	skull (cast)	adult	Hell Creek: MT
	*Pachycephalosaurus wyomingensis*	Sues & Galton (1987)				
DMNS 469	*Pachycephalosaurus reinheimeri*	Brown & Schlaijker (1943)	x	skull	adult	Lance: SD
	*Pachycephalosaurus wyomingensis*	Sues & Galton (1987)				
VRD 13	*Pachycephalosaurus wyomingensis*	Goodwin & Horner (2004)		skull	adult	Hell Creek: MT
UCMP 131334	*Pachycephalosaurus wyomingensis*	This study		skull	adult	Hell Creek: MT
MOR 295	Pachycephalosauridae indet.	This study		partial skull	younger adult	Hell Creek: MT
MOR 597	*Stegoceras* sp.	This study		?postorbital	juvenile-subadult	Judith River: MT

Published name and citation, type status, element, ontogenetic series, formation and state from the Upper Cretaceous of the Western Interior, USA, are listed. All formations are Maastrichtian age except for the Campanian Judith River Formation. Abbreviations: Fm, formation; lit., literature review only; MT, Montana; ND, North Dakota; SD, South Dakota; WY, Wyoming.

Histological examination of the cranial ornamentations of dinosaurs reveals complex morphologies composed primarily of tissue formed during metaplasia. Metaplasia is the process in which dense fibrous connective tissues are transformed directly into bone without the intervention of a periosteum or the presence of osteoblasts [23]. Metaplastic bone, particularly as seen in dinosaur crania, has a wide variety of forms, from highly porous to extremely dense. These tissues can appear very similar to bone deposited by a periosteum in that they contain vascular canals, osteocyte-like lacunae and can be reworked by Haversian-like innervations. Although similar in appearance, the structures that look like osteocytes lack canaliculi, and instead possess stubby lateral processes that do not connect to adjacent cells [24]. These structures are herein referred to as fibrocytes and apparently represent trapped fibroblasts. Metaplastic bone is common in reptiles [25], but generally described in association with osteoderms [25]–[29] and ossified tendons [30]. The metaplastic tissues in the skulls of pachycephalosaurids vary greatly in gross appearance depending on the cranial structure in which they form. As observed for other bone tissues, these metaplastic tissues undergo a consistent, predictable development in that the younger tissues are much more vascularized compared to increasingly mature tissues [17].

**Figure 8:**
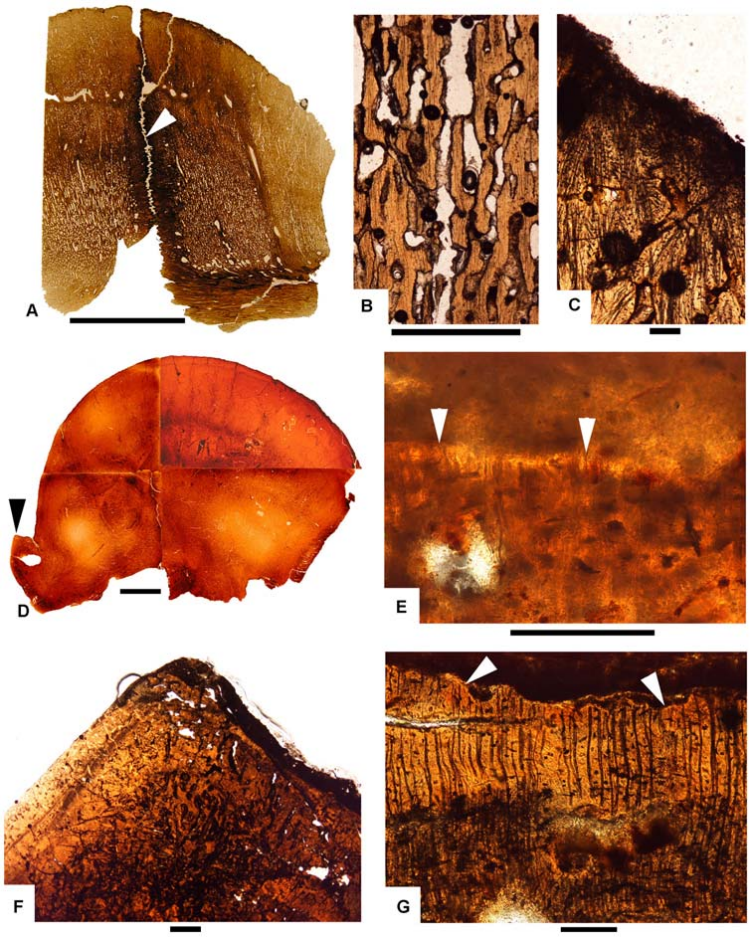
Craniohistological coronal sections of “*Stygimoloch*” (A–C) and *Pachycephalosaurus* (D–F) skulls. (A) UCMP 128383.PSF-3, a coronal section through the frontoparietal dome clearly shows the open intrafrontal suture (white arrow) in this subadult “*Stygimoloch*”. Scale bar is 2 cm. (B) UCMP 128383.PSF-3, a view of the middle region of the frontoparietal dome with highly vascularized tissue and vascular spaces oriented in a radial pattern. Scale bar is 1 mm. (C) UCMP 128383.PSF-3, the dorsal-most region of the frontoparietal dome reveals abundant fibers and fibrocytes present when the frontoparietal dome inflates and ossifies. Scale bar is 100 µm. (D) Coronal section of “*Pachycephalosaurus*”, VRD 13. The denser and less vascularized tissue throughout this late growth stage suggests subsidence of frontoparietal dome growth. Black arrow points to postorbital nodal ornament sectioned in (F) and (G). Scale bar is 2 cm. (E) VRD 13, the ends of fibers (white arrows) extend into the outermost margin of the interior of the dome and likely connected to a fibrous epidermal covering. Scale bar is 100 µm. (F) VRD 13, a postorbital nodal ornament in transverse section lacks any evidence of an epidermal attachment or growth dorsally. Scale bar is 1 mm. (G) VRD 13, the erosional surface (white arrows) points to continued modification in size and shape of this postorbital nodal ornament. No evidence of an epidermal attachment or growth dorsally in the degraded surface preserved in the overlying tissue. Scale bar is 100 µm.

A coronal histological section of a pachycephalosaurid skull previously referred to *“Stygimoloch”* (UCMP 128383) shows an open intrafrontal suture in coronal view (Figure 8A). The highly vascularized nature of the frontoparietal dome showing vascular spaces oriented primarily in a radial pattern (Figure 8B) suggests that the dome was actively growing at a rapid pace [22:512]. Abundant fibers and fibrocytes located at the dorsal surface of the dome indicate an area of apparent inflation and ossification (Figure 8C). A fully formed dome referable to *Pachycephalosaurus* (Figure 8D) is much denser with comparatively less vascularization suggesting that growth has subsided [17]. Extending over the entire outer-most margin of the dome interior are the ends of fibers that apparently connected to a fibrous epidermal covering the dome (Figure 8E). The presence of these exposed fibers suggests the dome was still inflating as there is no evidence of an erosional surface. A node on the postorbital bar (Figure 8F), by contrast, possesses a surface that is erosional (Figure 8G), blunt-shaped and lacks any evidence of attachment or growth. An overlying matrix preserves this degraded surface.

The most interesting and controversial aspect of our hypothesis, that “*Dracorex*” and “*Stygimoloch*” represent younger ontogenetic stages of *Pachycephalosaurus*, relates to the squamosal horns. They are medium to large pointed structures in “*Dracorex*” [20] and “*Stygimoloch*” (Figure 1, Figure 2), but are relatively shorter, blunt to lightly pointed and robust in *Pachycephalosaurus* (Figure 6, Figure 7). Three squamosal horns were sectioned to establish this sequence, including the holotype of *“Stygimoloch”* (UCMP 119433). If *“Dracorex”* and “*Stygimoloch*” represent ontogenetic stages of *Pachycephalosaurus*, then it must be shown that these horns grow and elongate in *“Dracorex”* and *“Stygimoloch”* before the onset of erosion when they are reduced in size and modified in shape. We do not know at what growth stage maximum squamosal horn length is achieved, but it appears to occur between the holotype of *“Dracorex”* and some stage of *“Stygimoloch”*.

**Figure 9:**
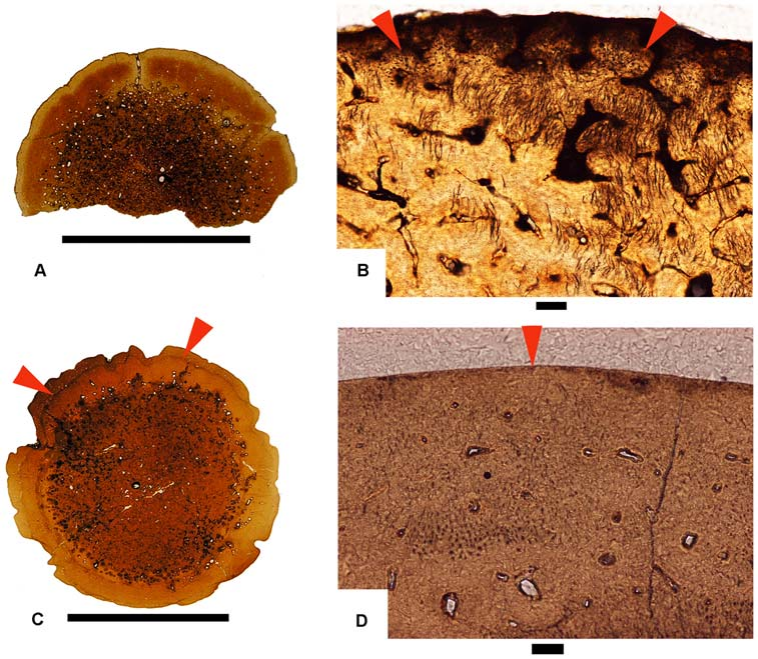
Histological sections of “*Stygimoloch*” squamosal horns. (A) UCMP 128383.CrSp.1–2, transverse section of a highly-vascularized, expanding “*Stygimoloch*” squamosal horn found associated with a frontoparietal dome (see Fig. 3A) and partial skeleton. Scale bar is 2 cm. (B) UCMP 128383.CrSp.1–2, the red arrows point to the interior depositional surface and expansion of this squamosal horn. This metaplastic tissue closely resembles bone that contacts a periosteum, but the absence of osteocytes and abundant fibers indicates the horn is expanding through metaplasia rather than periosteal ossification. Scale bar is 100 µm. (C,D) UCMP 119433.SqSp.1–4, transverse sections reveal metaplastic tissue in the largest squamosal horn from the holotype of “*Stygimoloch*” (UCMP 119433). In (C), the interior erosional line is interrupted by a depositional surface along the red arrows. Scale bar is 2 cm. In (D), the erosional surface (red arrow) indicates the horns are getting smaller. Scale bar is 100 µm.

The smallest horn (UCMP 128383) was found associated with the *“Stygimoloch”* dome in Figure 8A, revealing evidence of rapid dome growth. This horn also reveals highly vascularized tissue (Figure 9A) indicative of rapid expansion in size and an extremely irregular exterior surface (Figure 9B) that is very similar to bone that contacts a periosteum. The absence of osteocytes and abundance of fibers indicates the bone is expanding through metaplasia rather than periosteal ossification. A cross section through the largest central horn (#1 sensu Galton and Sues [31]) from the holotype of *“Stygimoloch”* (Figure 1A) reveals metaplastic tissue with an embayed exterior surface (Figure 9C) that is being eroded (Figure 9D). There is no evidence that metaplasia was occurring at the time of the animal's death; however, there is an indication histologically that the horn underwent an earlier period of erosion followed by a phase of metaplasia, and subsequently a final period of erosion that was occurring when the animal died. Evidence for this sequence is the erosional unconformity indicated by the arrows in Figure 9C.

**Figure 10:**
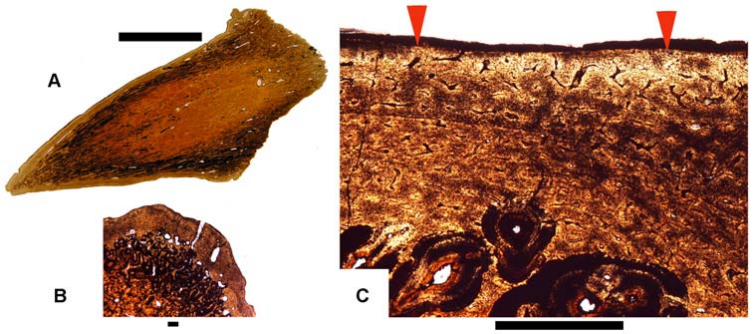
Histological sections of “Stygimoloch” squamosal horn. (A–C), MOR 560.CrSp.1-L2, a squamosal horn in erosion mode in (A) longitudinal and (B,C) transverse section becomes smaller. The red arrows in (C) point to a surface that is degrading with no sign of metaplasia with a thin metaplastic veneer. Scale bar is 2 cm in (A) and 1 mm in (B) and (C).

Another horn (MOR 560; found isolated) cut both longitudinally (Figure 10A) and transversely (Figure 10B) is also in an erosion mode as evidenced by a surface that is degrading with no signs of metaplasia (Figure 10C). The primary metaplastic veneer is very thin, and most of the bone has been reconstructed by secondary innervations. Sharpey's fibers near the exterior surfaces and indented vessel channels on the exterior surfaces of each of the sectioned horns suggest that they were keratin covered regardless of whether they were undergoing metaplasia or erosion.

### Conclusions

We propose that *Dracorex hogwartsia* and *Stygimoloch spinifer* are growth stages of *Pachycephalosaurus wyomingensis* and represent an ontogenetic series of *P. wyomingensis* united by shared morphology and increasing skull length. The youngest and most complete skull of *Pachycephalosaurus* yet known belongs to the growth stage best illustrated by *“Dracorex”*. This synonymy significantly reduces the number of Upper Cretaceous pachycephalosaurid taxa.

Timing of dome inflation is probably highly variable based on the cranial patterns preserved in *Stegoceras* [4], [32] and *Triceratops* [15], [16]. Like some hadrosaurids and ceratopsids, these pachycephalosaurids retain their juvenile cranial characters until they are at least 50% grown. This ontogenetic pattern contributed to the naming of some new pachycephalosaurid taxa based on diagnostic characters (large supratemporal fenestrae, squamosal horns, nodal cranial ornamentation, inflated frontoparietal dome) that we demonstrate in this study are, in fact, ontogenetic features that undergo extreme modification as the skull length increases, the cranial vault enlarges and the frontoparietal dome expands.

The squamosal horns and nodes of *Pachycephalosaurus* underwent a similar transformation to the epiparietal and episquamosal elements of *Triceratops* [16:figure 2] that (1) grew from diminutive to large triangular-shaped ornaments; (2) eroded as they reduced dorsoventrally and (3) flattened and lengthened as they merged onto the edge of the parietal-squamosal frill. Pachycephalosaurid horns originated ontogenetically as node-like structures, some of which expanded into horns and bony spikes and then became modified as blunt, nodal structures in late stage ontogeny. The various shapes of the frontoparietal dome, from the characteristic tall broad dome of the *Pachycephalosaurus wyomingensis* (AMNH 1696) to the relatively flattened morphology of *Pachycephalosaurus “reinheimeri”* (DMNS 469) also reflect a comparable morphological transformation that occurs ontogenetically.

Marginocephalian dinosaurs employ metaplasia to grow their horns, cranial ornaments, domes and shields as they rapidly remodel their skulls. Any hypothesis testing of head butting in pachycephalosaurids, combat in ceratopsids or similar agonistic encounters must take into account the fact that the skulls of these dinosaurs are composed largely of metaplastic tissue.

## Materials and Methods

Twenty-one pachycephalosaurid skulls and cranial elements examined in this study are listed in Table 2. Four casts were included to confirm external morphologies and supplement fossil specimens used for histological analysis. Cut blocks of fossils were embedded in Silmar-40 polyester resin prior to histological sectioning and mounted to glass slides with epoxy resin. Archival casts were made of the specimens before cutting and are catalogued in the collections of the MOR and UCMP. The thin sections were ground on a Buehler Ecomet grinder and sections were studied by light microscopy under normal and polarized light. Histological sections from this study are catalogued into the slide libraries of the MOR and UCMP. Some of the histological slides were prepared for a previous study by the authors [17].

## Supporting Information

Text S1 Historical Review and Systematic Paleontology of *Pachycephalosaurus*
(0.05 MB DOC)
