# Supplementary material for: Extreme Cranial Ontogeny in the Upper Cretaceous Dinosaur Pachycephalosaurus
Source: PLoS One. 2009 Oct 27;4(10):e7626. doi: 10.1371/journal.pone.0007626 (PMC2762616; doi:10.1371/journal.pone.0007626)
Supplement: Text S1 — Historical Review and Systematic Paleontology of Pachycephalosaurus (0.05 MB DOC) [file pone.0007626.s001.doc]

**SUPPORTING INFORMATION**

The history of the genus *Pachycephalosaurus* involves many prominent dinosaur paleontologists of the early 20^th^ century. In 1931, Charles Gilmore [S1] referred a posterior partial dinosaur skull (USNM 12031) from the Lance Formation of Niobrara (=Converse) County, Wyoming, to the genus *Troödon* and the new species, *T. wyomingensis*. Gilmore cited John Bell Hatcher’s 1905 account [S2] of the occurrence of *Troödon* teeth in the Laramie (=Lance) Formation, Converse (=Niobrara) County, Wyoming, as corroboration for his identification of this enlarged dinosaur skull as *Troödon*. Previously in 1924, Gilmore [S3] described the first skull and partial skeleton of *Troödon* *validus* (=*Stegoceras validum*) and was therefore familiar with the distinctive frontoparietal dome when he described USNM 12031. Gilmore [S1:2] observed, “The cranium-shaped enlargement is the outstanding feature of the *Troödon* skull…” and noted that the smooth, large size of the dome may be “…only an age characteristic.”

In 1943, Brown and Schlaikjer [S4] published their monograph on troödont dinosaurs. They named a new genus, *Pachycephalosaurus*, and included three species, two of which were new: *P. grangeri* (AMNH 1696; a nearly complete skull); and *P. reinheimeri* (DMNS 469; a frontoparietal dome). They considered *Troödon wyomingensis* Gilmore 1931 a junior synonym of *Pachycephalosaurus wyomingensis*. The diagnostic features listed by Brown and Schlaikjer [S4:132] for the skull of *Pachycephalosaurus* are: extreme thickening of the frontoparietal area; node-like ornamentation strongly developed; supratemporal openings completely closed; face narrow and shallow. Diagnostic characters for the three species of *Pachycephalosaurus* are summarized here from Gilmore [S1], Brown and Schlaikjer [S4] and Galton and Sues [S5]: *P. grangeri*: frontoparietal high, arched and greatly thickened; lacks a parietosquamosal shelf; frontals deep and narrow at contact with nasals; occipital condyle positioned forward; *P. wyomingensis* (Gilmore 1931): Gilmore’s 1931 study [S1] lacks a proper diagnosis but the following features are included in his detailed description of the type skull, USNM 12031 and comparison to known specimens of “*Troödon validus*” at the time: large fused cranium; supratemporal fenestrae closed; dome surface smooth, unsculptured and ornamentation reduced; steeply inclined parietal posterior to the dome; and *P. reinheimeri*: frontoparietal very low, broad and shallow; prominent parietosquamosal shelf with nodal ornamentation and deeply notched medially; frontals low and narrow at contact with the nasals.

In 1945, Sternberg [S6] proposed the name Pachycephalosauridae for the dome-headed dinosaurs based, in part, on characters presented by Gilmore [S3:8] for the Troödontidae. Sternberg [S6:534] asserted that the name *Pachycephalosaurus* rather than *Troödon* “...happily stresses the thick-skulled character of the animal.”

A review of North American members of the Pachycephalosauria by Sues and Galton [S7] recognized *Pachycephalosaurus* Brown and Schlaikjer 1943 as a valid genus with only a single species, which by priority [S1] is *P. wyomingensis*. Sues and Galton [S7] argued that the diagnostic characters for *P. wyomingensis* (Gilmore 1931) and *P. grangeri* and *P. reinheimeri* Brown and Schlaikjer 1943 are highly variable. In their review, Sues and Galton listed the following cranial characters: *P. grangeri* Brown and Schlaikjer 1943 (AMNH 1696) possesses an extremely large dome and lacks a parietosquamosal shelf. *P. wyomingensis* (Gilmore 1931) and *P. reinheimeri* Brown and Schlaikjer 1943 have a relatively low frontoparietal vault and a strong parietosquamosal shelf. The sutural contact between the nasal and frontal is narrow in the type skulls of *P. grangeri* (AMNH 1696) and *P. reinheimeri* (DMNS 469) and wide in *P. wyomingensis* (USNM 12031). We accept this synonymy by Sues and Galton [S7] as do most students of pachycephalosaurids.

Galton and Sues [S5] named *Stygimoloch spinifer* (UCMP 119433) from an unusual partial left squamosal from the Hell Creek Formation, McCone County, Montana. This squamosal is ornamented by “three or four massive horn-cores” with a prominent shelf and open supratemporal fenestrae (in juveniles). In the same study, Galton and Sues referred YPM 335, previously identified as a dermal ossification of *Triceratops* by Marsh [S8] and refigured by Hatcher et al. [S9], to *S. spinifer*. Brown and Schlaikjer [S4] referred YPM 335 to *Pachycephalosaurus* sp. in their description of a new “troödont” (=pachycephalosaurid) genus and four new species. Brown and Schlaikjer [S4:45] stated, “This specimen [YPM 335] may well represent a new species of *Pachycephalosaurus*” but presciently cautioned, “…a new species should not be founded on such fragmentary material.”

Giffin et al. [S10] described a tall, narrow-domed pachycephalosaurid skull (MPM 7111) from the Hell Creek Formation of eastern Montana as a new genus and species, *Stenotholus kohleri*, primarily on the basis of the shape of the frontoparietal dome. Gabriel and Berghaus [S11] synonymized *S. kohleri* with *Stygimoloch spinifer* upon the discovery of MPM 8111, a more complete pachycephalosaurid skull with a distinctive horn-covered articulated squamosal. Gabriel and Berghaus [S11] recognized that both MPM 7111 and 8111 shared the relatively high, narrow frontoparietal dome and cranial features previously attributed to *S. spinifer*. Goodwin et al. [S12] provided an extensive description of the cranial anatomy of *S. spinifer* based on MPM 8111 and previously undescribed skulls and cranial specimens from the Hell Creek Formation, Montana and the contemporaneous Lance Formation, Wyoming. Williamson and Carr [S13] referred a fragmentary pachycephalosaurid frontoparietal (TMP 81.27.24) from the Campanian Dinosaur Park Formation, southern Alberta, to *Stygimoloch* sp. They suggested that the preservation of open temporal fenestrae and a distinct frontoparietal might represent an early cranial growth stage.

Bakker et al. [S14] described *Dracorex* *hogwartsia* (TCNI 2004.17.1) as a “young-adult” pachycephalosaurid from the Upper Cretaceous Hell Creek Formation (Lancian) of South Dakota on the basis of a nearly complete skull and the third, eighth and ninth cervical vertebrae. *D. hogwartsia* is 42 cm long along the midline, lacks the characteristic frontoparietal dome and has unusually large supratemporal fenestrae compared to any other pachycephalosaurid. Bakker et al. [S14] proposed that the opened supratemporal fenestrae are juvenile features retained in the adult skull. Lack of a frontoparietal dome is hypothesized to be phylogenetically derived, indicative of a character reversal or paedomorphosis, not ontogeny. The anterior and posterior supraorbitals and postorbitals 1 and 2 are suturally distinct and observable on the skull. According to the authors, these “primitive” pachycephalosaurid cranial features retained by *D. hogwartsia* are unexpectedly preserved with more derived features, such as the “large *Stygimoloch*-style squamosal spikes, large cheek nodes (‘hornlets’) and a long snout, with prominent pyramidal nodes also seen in *Pachycephalosaurus*” [S14:331].

Systematic Paleontology

Family Pachycephalosauridae Sternberg, 1945

*Pachycephalosaurus wyomingensis* Gilmore, 1931, p. 1, pls. 1,2,3; Sues and Galton, 1987, p. 29, fig. 16.

*Dracorex hogwartsia* Bakker, Sullivan, Porter, Larson and Saulsbury, 2006, p. 331.

*Stygimoloch spinifer* Galton and Sues, 1983, p. 463, fig. 3; Galton and Sues, 1987, p. 33, pl. 7, figs. 1-5; Gabriel and Berghaus, 1988, p. 10; Giffin, Gabriel and Johnson, 1988, figs. 2, 3, 4, 5, 6; Goodwin, Buchholtz and Johnson, 1998, figs. 1-21.

*Occurrence–*Upper Cretaceous (Maastrichtian and/or Lancian equivalent strata), Western Interior of North America.

*Discussion–*We follow the diagnosis of *Pachycephalosaurus* *wyomingensis* of Sues and Galton [7] and their recommendation that all three previously described species of *Pachycephalosaurus* from the Western Interior of North America (*P. grangeri* Brown and Schlaikjer 1943 and *P. reinheimeri* Brown and Schlaikjer 1943 are subjective junior synonyms) represent the single taxon, *P. wyomingensis* [S1]. We do not agree with their conclusion that the skulls of *P. wyomingensis*, represented by AMNH 1696 and DMNS 469, are male and female dimorphs respectively. These skulls are not from the same locality or assemblage, and gender assignment cannot be effectively tested at this time. Alternatively, DMNS 469 may represent a very mature individual.

References for Supporting Information Text S1

S1. Gilmore CW (1931) A new species of Troödont dinosaur from the Lance Formation of

Wyoming. Proc US Nat Mus 79: 1-6.

S2. Stanton TW, Hatcher JB (1905) Geology and Paleontology of the Judith River Beds. US

Geol Surv Bull 257: 1-128.

S3. Gilmore CW (1924) On *Troödon validus*, an ornithopodous dinosaur from the Belly River

Cretaceous of Alberta, Canada. Univ Alberta Bull 1: 1-43.

S4. Brown B, Schlaikjer EM (1943) A study of the Troödont dinosaurs with the description of a

new genus and four new species. Bull Am Mus Nat Hist 82: 121-149.

S5. Galton PM, Sues H-D (1983) New data on pachycephalosaurid dinosaurs

(Reptilia: Ornithischia) from North America. Canadian Journal of Earth Sciences. 20: 462-472.

S6. Sternberg CM (1945) Pachycephalosauridae proposed for dome-headed dinosaurs,

*Stegoceras lambei*, N. Sp., described. J Paleont 19: 534-538.

S7. Sues H-D, Galton PM (1987) Anatomy and classification of the North

American Pachycephalosauria (Dinosauria: Ornithischia). Palaeont Abt A 198: 1-40.

S8. March OC (1896) The dinosaurs of North America. Sixteenth Annual Report, US

Geol Surv 1894-95 Part 1: 133-414.

S9. Hatcher JB, Marsh OC, Lull RS (1907) The Ceratopsia. US Geol Surv Mon 49: 1-300.

S10. Giffin EB, Gabriel DL, Johnson RE (1987) A new pachycephalosaurid skull (Ornithischia) from the Cretaceous Hell creek Formation of Montana. J Vert Paleont 7: 398-407.

S11. Gabriel DL, Berghaus CB (1988) Three new specimens of *Stygimoloch* *spinifer* (Ornithischia; Pachycephalosauridae) and behavioral inferences based on cranial morphology. In: International Symposium on Vertebrate Behavior as Derived from the Fossil Record. Bozeman: Museum of the Rockies, Montana State University, Montana. pp. 10.

S12. Goodwin MB, Buchholtz EA, Johnson RE (1998) Cranial anatomy and diagnosis

of Stygimoloch spinifer (Ornithischia: Pachycephalosauria) with comments on cranial display structures in agonistic behavior. J Vert Paleont 18: 363-375.

S13. Williamson TE, Carr TD (2006) First record of a Pachycephalosaurine (Ornithischia:

Pachycephalosauria) from the Dinosaur Park Formation, Alberta, Canada. In: Lucas, SG, Sullivan, RM, editors. Late Cretaceous vertebrates from the Western Interior. New Mexico Museum of Natural History and Science Bulletin 35. pp. 323-327.

S14. Bakker RT, Sullivan RM, Porter V, Larson P, Saulsbury SJ (2006) *Dracorex hogwartsia*, n.

gen., n. sp., a spiked, flat-headed pachycephalosaurid dinosaur from the Upper Cretaceous Hell Creek Formation of South Dakota. In Lucas SG, Sullivan RM, editors. Late Cretaceous vertebrates from the Western Interior. New Mexico Museum of Natural History and Science Bulletin 35. pp. 331-345.
